# Type 2 Diabetes Remission and Substantial Body Weight Reduction Achieved with Metformin and a Sodium-Glucose Cotransporter 2 Inhibitor

**DOI:** 10.7759/cureus.7110

**Published:** 2020-02-26

**Authors:** Seigo Sugiyama, Hideaki Jinnouchi, Kunio Hieshima, Noboru Kurinami, Katsunori Jinnouchi

**Affiliations:** 1 Diabetes Care Center, Jinnouchi Hospital, Kumamoto, JPN

**Keywords:** diabetes remission, types 2 diabetes, weight reduction, insulin resistance, sodium-glucose cotransporter 2 inhibitor

## Abstract

The overall goal in the treatment of type 2 diabetes mellitus (T2DM) is remission. However, the effects of a sodium-glucose cotransporter 2 inhibitor (SGLT2i) on remission of T2DM are unknown. We herein report a case involving an overweight 43-year-old man who completely recovered from T2DM after SGLT2i therapy (dapagliflozin at 5 mg/day). In the pretreatment period, he had a body mass index (BMI) of 26.0 kg/m^2^, hemoglobin A1c (HbA1c) concentration of 10.3%, advanced insulin resistance, pancreatic β-cell dysfunction, and fatty liver. Eighteen months after comprehensive therapy, including the administration of an SGLT2i and metformin, his BMI had decreased to 21.3 kg/m^2^ and his glycemic control was almost normal (HbA1c of 5.3%) despite discontinuation of all hypoglycemic medications. This report is the first to propose the usefulness of the combination therapy of SGLT2i and metformin for achieving normal body weight and remission of newly diagnosed T2DM in a real-world clinical situation.

## Introduction

Type 2 diabetes mellitus (T2DM) is widely recognized as a chronic progressive disease that requires lifelong hypoglycemic treatment [1]. However, some patients can maintain good glycemic control by only diet and exercise therapy after receiving a definitive diagnosis of T2DM [2]. T2DM has various pathogenic causes and is associated with several clinical conditions, and the disease follows diverse clinical courses among affected patients [3].

The onset of T2DM is strongly associated with a gain in body weight and excess ectopic fat accumulation in the liver and pancreas [4-5]. During the early phase of T2DM, lifestyle modification by diet and exercise therapy is recommended to achieve appropriate body weight and caloric intake. If adequate glycemic control is not achieved, the addition of glucose-lowering pharmacotherapy is considered [2]. However, the provision of specific and personal direction and instruction for lifestyle improvement tends to be insufficient in daily clinical practice. The enforcement and continuation of a strict diet and exercise therapy are often difficult in the food-infatuated daily life situation of many patients with T2DM [6].

Sodium-glucose cotransporter 2 inhibitors (SGLT2is), which are effective glucose-lowering drugs, reduce the blood glucose concentration by increasing urinary glucose excretion in an insulin-independent manner. This results in the metabolism of the accumulated fat and a reduction in body weight by loss of calories into the urine [7]. This SGLT2i-induced weight loss might be beneficial for a wide range of patients with T2DM [8-9].

We herein describe a patient with T2DM in whom the hemoglobin A1c (HbA1c) concentration successfully decreased to almost a normal level with substantial weight loss after comprehensive therapy, including administration of metformin and SGLT2i (dapagliflozin at 5 mg/day). Our patient completely discontinued all hypoglycemic drugs, leading to remission of T2DM [10].

## Case presentation

In early April 2018, a 43-year-old man presented to the Diabetes Care Center at Jinnouchi Hospital in Kumamoto, Japan, because of the inadequate control of T2DM. At 33 years of age, he had been diagnosed with obesity (body mass index (BMI) of 28.7 kg/m^2^), sleep apnea syndrome, and hypertension. At that time, he was treated with continuous positive airway pressure, an angiotensin II receptor blocker, a calcium channel antagonist, and a thiazide diuretic by his primary care physician. Two months before his initial visit to our hospital, he developed general fatigue, weight loss (from 86 to 81 kg), and lower limb cramps during his work as a salesman. He did not have a habit of excessive soft drink intake. His symptoms did not improve, and an elevated fasting serum glucose concentration of 252 mg/dL was first detected at an annual health check-up in March 2018. He also had a strong family history of T2DM (grandmother, father, and brother). He became seriously concerned about his clinical condition and decided to visit our Diabetes Care Center.

At his first visit to our outpatient service, clinical examination showed a body height of 174 cm, body weight of 80.8 kg, BMI of 26.0 kg/m^2^, blood pressure of 118/65 mmHg, and regular pulse rate of 94 beats/min. Physical examination revealed no abnormalities. Laboratory examination showed hyperglycemia (fasting blood glucose concentration, 157 mg/dL); elevated concentrations of HbA1c (10.3%), aspartate transaminase (38 IU/L), and alanine transaminase (46 IU/L); and a reduced estimated glomerular filtration rate (68.1 mL/min/1.73 m^2^). Furthermore, the patient had proteinuria (±), hematuria (+), and positive urinary ketone bodies (+) (Table 1).

**Table 1:** Laboratory data at initial visit to Jinnouchi Hospital AST: aspartate aminotransferase, ALT: alanine aminotransferase, γGTP: γ glutamyl transpeptidase, LDH: lactate dehydrogenase, ALP: alkaline phosphatase, CPK: creatinine phosphokinase, BUN: blood urea nitrogen, HDL: high-density lipoprotein, LDL: low-density lipoprotein, MCV: mean corpuscular volume, MCH: mean corpuscular hemoglobin, MCHC: mean corpuscular hemoglobin concentration

Biochemistry		Glucose metabolism	
Total protein (g/dL)	7.2	Fasting blood glucose (mg/dL)	157
Albumin (g/dL)	4.6	Hemoglobin A1c (%)	10.3
Total bilirubin (mg/dL)	0.9		
AST (IU/L)	38	[Blood cell count]	
ALT (IU/L)	46	White blood cells (/mL)	4670
g-GTP (IU/L)	30	Red blood cells (/mL)	493 x 10^4^
LDH (IU/L)	158	Hemoglobin (g/dL)	15.2
ALP (IU/L)	184	Hematocrit (%)	44.7
CPK (IU/L)	246	MCV (fL)	90.7
Amylase (IU/L)	52	MCH (pg)	30.8
Total-cholesterol (mg/dL)	192	MCHC (%)	34.0
HDL-cholesterol (mg/dL)	41	Platelets (/mL)	25.7 x 10^4^
Triglyceride (mg/dL)	78		
LDL-cholesterol (mg/dL)	135	[Urinary data]	
BUN (mg/dL)	11.2	pH	6.0
Creatinine (mg/dL)	0.89	Specific gravity	1.025
Uric acid (mg/dL)	6.8	Protein	+/-
Sodium (Na; mEq/L)	137	Glucose	-
Potassium (K; mEq/L)	4.2	Occult blood	+
Chloride (Cl; mEq/L)	98	Ketone	+
Calcium (Ca; mg/dL)	9.3	Urobilinogen	+/-

He did not have diabetic retinopathy, neuropathy, thyroid dysfunction, adrenal abnormality, or a history of cardiovascular disease.

A few days after his first visit, he was admitted to our hospital for the assessment and treatment of his T2DM. We found that his antibody titer to glutamic acid decarboxylase was undetectable (<5.0 U/mL). Additionally, his urinary C-peptide immunoreactivity excretion was well preserved at 96.5 µg/day, thus establishing a definitive diagnosis of T2DM. On the third day of hospitalization, a hyperinsulinemic-euglycemic clamp examination revealed insulin resistance (Table 2) [11].

**Table 2:** Baseline assessment of diabetic condition and diabetes-associated complications CPR: C-peptide immunoreactivity, GAD: glutamic acid decarboxylase, M-value: glucose infusion rate, M/I: (M-value)/(steady state insulin), eGFR: estimated glomerular filtration rate, CT: computed tomography, PAT: peripheral arterial tonometry (index of microvascular endothelial function)

	Measured Values/Finding	Assessment
Family history of diabetes	Grandmother, father, and brother	Present
Maximum body weight at 39 years old (kg)	89	-
Maximum body mass index (kg/m^2^)	28.7	Overweight
Fasting CPR (ng/mL)	2.18	Normal
Urinary CPR excretion (mg/day)	96.05	Normal
Anti-GAD antibody (U/mL)	< 5.0	Normal
Fasting glucagon (pg/mL)	145	Elevated
Post-prandial glucagon (pg/mL)	143	No suppression
Euglycemic Hyperinsulimenic Clamp		
M-value (mg/kg/minute)	6.35	Low
M/I (g•L/U/kg/minute)	5.92	Low
Steady-state insulin (mU/mL)	107.2	Normal
Retinopathy	None	Absent
Urinary albumin excretion (mg/day)	6.6	Normal
eGFR (mL/min/1.73m^2^)	75.0	Stage-2
Achilles tendon reflex	Right: +, Left: +	Normal
Abdominal ultrasound	Liver high echo, gallbladder polyp	Fatty liver
Visceral fat area in abdominal CT (cm^2^)	81.9	Normal
Carotid artery ultrasound	Plaque in left carotid bulb (1.97 mm)	Atherosclerosis
Reactive hyperlemia-PAT	1.60	Impaired

**Figure 1:**
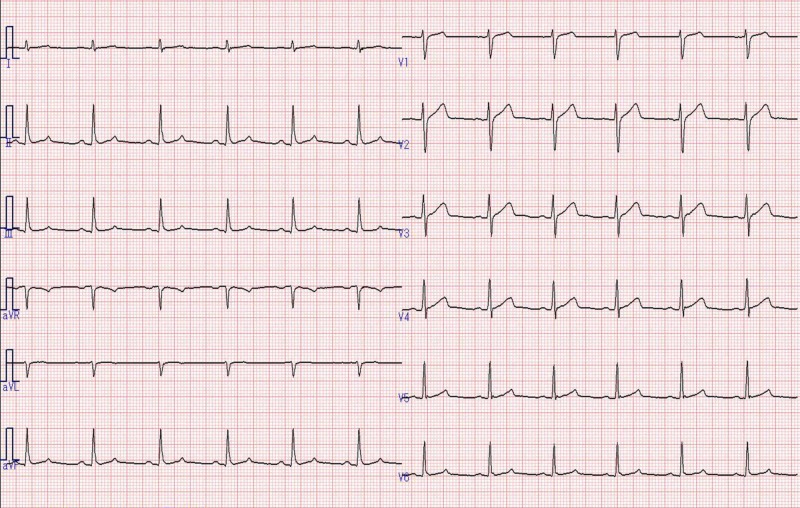
Electrocardiogram

**Figure 2:**
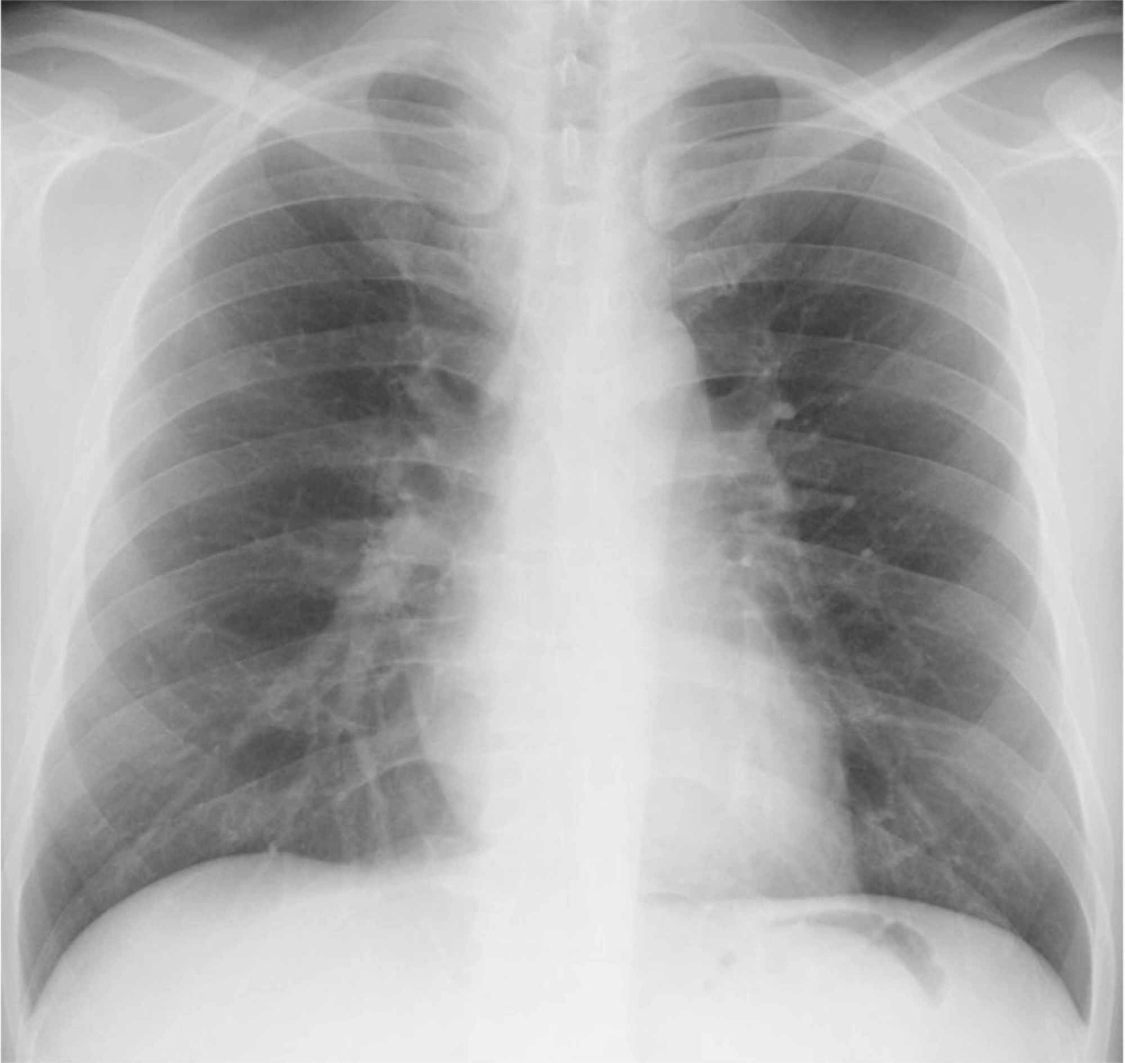
Chest radiograph

An electrocardiogram (Figure 1) and chest radiograph (Figure 2) revealed no abnormalities.

**Figure 3:**
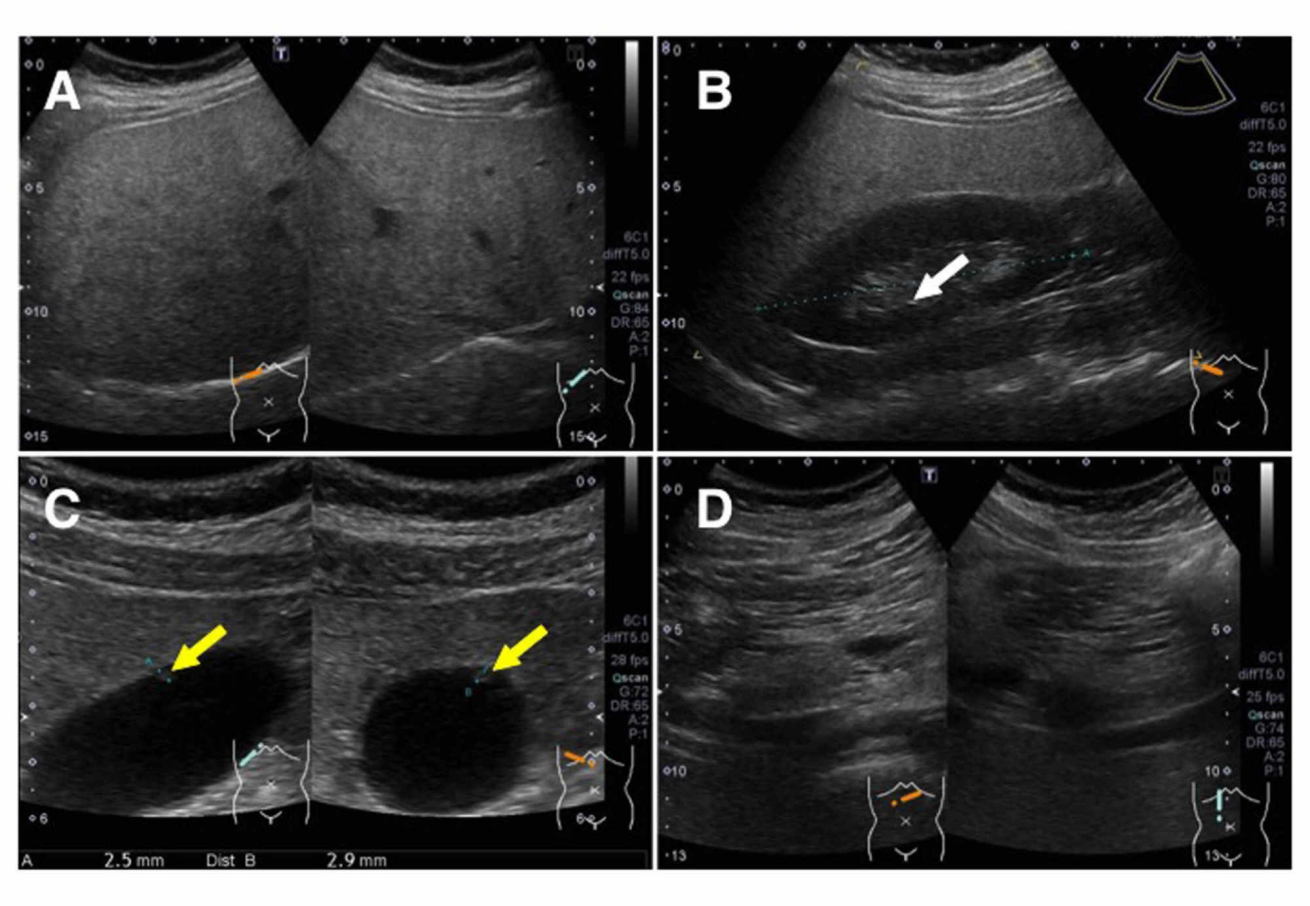
Abdominal ultrasound images before therapy A: Liver images; B: Liver and right kidney image, white arrow indicates kidney calcification; C: gallbladder image, yellow arrows indicated a gallbladder polyp; D: pancreas images

An abdominal ultrasound examination showed fatty liver (Figure 3A-3B), right renal calcification (Figure 3B, a white arrow), a gallbladder polyp (Figure 3C, yellow arrows), and no abnormalities in the pancreas (Figure 3D).

**Figure 4:**
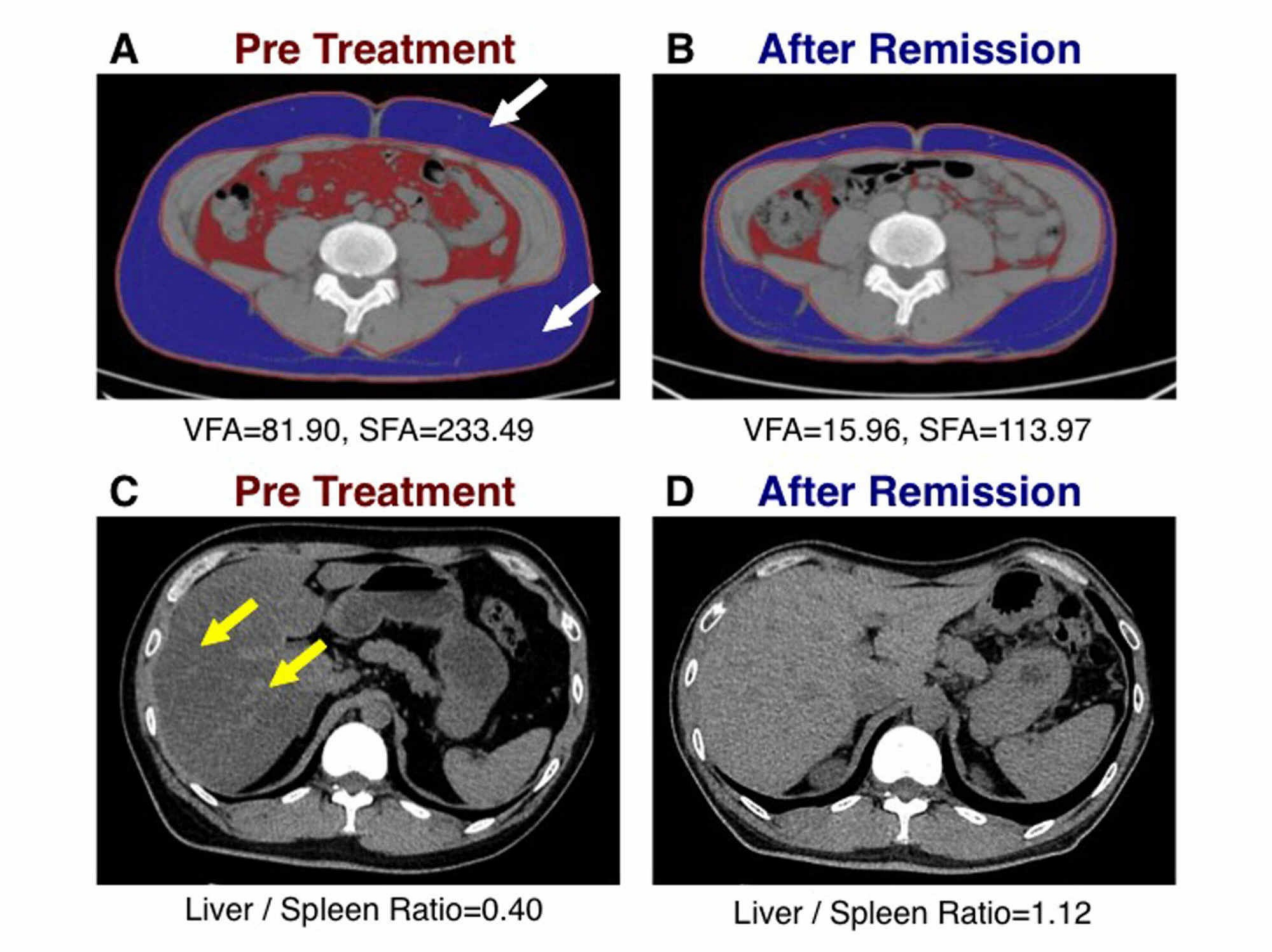
Changes in abdominal and liver fat accumulation after remission of diabetes Fat scan images and abdominal computed tomography images before therapy and after remission of diabetes. Blue areas indicated subcutaneous fat and red areas indicated visceral fat assessed by fat scan; VFA: visceral fat area (cm^2^), SFA: subcutaneous fat area (cm^2^). White arrows indicate the abdominal subcutaneous fat accumulation and yellow arrows indicate fatty liver. A: Fat scan image before therapy; B: Fat scan image after remission; C: Liver and spleen image before therapy; D: Liver and spleen image after remission

Abdominal computed tomography showed excess subcutaneous fat deposition (Figure 4, white arrows) and increased hepatic fat accumulation (Figure 4, yellow arrows) as assessed by the liver-to-spleen attenuation ratio (Table 3) [12].

**Table 3:** Changes in glucose metabolic and anthropometric parameters HOMA-IR: Homeostasis model assessment for insulin resistance; HOMA-b: Homeostatic model assessment for beta cell function; QUICKI: Quantitative insulin sensitivity check index; M-value: Glucose infusion rate

	Pre-Treatment	Post-Treatment and Remission
Hemoglobin A1c (%)	10.3	5.3
Fasting plasma glucose (mg/dL)	157	93
Fasting Insulin (mU/mL)	8.7	2.5
HOMA-IR	3.37	0.57
HOMA-b	33.3	30.0
QUICKI	0.121	0.411
Insulinogenic index	0.03	0.75
Matsuda & DeFronzo index	3.25	10.84
Disposition Index	0.03	8.18
M-value (mg/kg/minute)	6.35	-
Body weight (kg)	80.8	66.5
Body mass index (kg/m^2^)	26.0	21.3
Waist circumstance (cm)	90.8	80.0
Body fat mass (kg)	22.2	8.8
Body fat percentage (%)	27.8	13.3
Skeletal muscle mass (kg)	32.8	32.0
Total body water (L)	42.5	42.5
Abdominal visceral fat area (cm^2^)	81.90	15.96
Abdominal subcutaneous fat Area (cm^2^)	233.49	113.97
Total abdominal fat area (cm^2^)	315.39	129.93
Liver/spleen attenuation ratio	0.40	1.12

His clinical condition suggested the possibility of hereditary factors for T2DM, pancreatic β-cell dysfunction, fatty liver, and insulin resistance with excess body weight and ectopic fat accumulation.

Because of the existing insulin resistance and the preserved capacity of intrinsic insulin secretion, the patient received lifestyle modification therapy during hospitalization. He was given printed information regarding the use of diet and exercise to treat T2DM. He also received an explanation of his present overweight condition and a target body weight of <70 kg. Instructions regarding dietary therapy were provided to the patient and his wife. On each day of hospitalization, the patient received managed hospital meals (1760 kcal/day based on 26 kcal/kg of ideal body weight; carbohydrates, 50%; protein, 1.2 g/kg; lipids, ≤300 mg/day cholesterol; non-saturated fatty acids, <10%; saturated fatty acids, <7%; no alcohol; and sodium chloride, 6 g/day). In-hospital exercise involved using treadmills and bicycle ergometers in the hospital exercise room and walking around the hospital. We provided instructions regarding moderate-intensity physical exercise, including resistance training, during each 30-minute session. After admission, his systolic blood pressure dropped to <100 mmHg. Therefore, we stopped all anti-hypertensive medications. He was initially treated with metformin (1000 mg/day) and subsequently with an additional SGLT2i (dapagliflozin at 5 mg/day), and his blood glucose concentration was well-controlled on the sixth day of hospitalization. After seven days of in-hospital therapy and education, the patient was discharged with the recommendation to continue appropriate dietary therapy (1760 kcal/day as described above) and at least 30 minutes of daily exercise. We explained that remission of T2DM would be possible after achieving an ideal body weight (<70 kg, which was his body weight at 20 years of age). The patient continued treatment with metformin and dapagliflozin and underwent monthly outpatient follow-ups.

**Figure 5:**
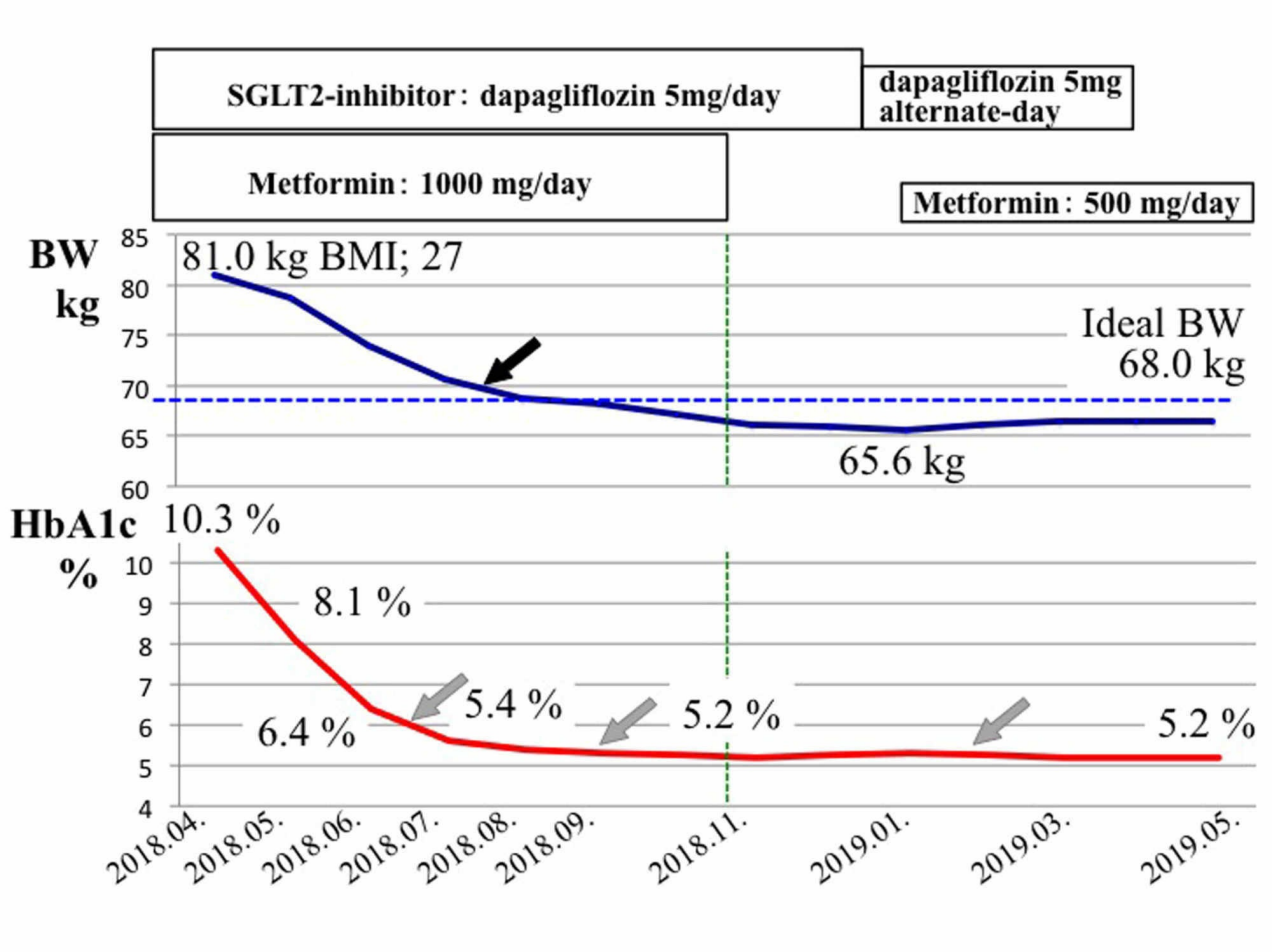
Time course of changes in body weight and HbA1c during post-discharge outpatient care BMI: body mass index; BW: body weight; HbA1c: hemoglobin A1c; SGLT2: sodium-glucose cotransporter-2 Black arrow indicates achieving a body weight of 70 kg, gray arrows indicate sustained good control of diabetes, blue dotted line indicates the level of ideal body weight, and green dotted line indicates the time point (November 2018) of normalization of both HbA1c and body weight

After discharge, he spontaneously began a 30-minute walking habit on the way to his workplace. He recorded his daily exercise achievements, blood pressure, pulse rate, and body weight in his outpatient care logbook. He successfully continued the dietary and exercise therapy and medications, which led to a gradual decrease in his body weight and HbA1c concentration. In November 2018, we temporarily stopped the metformin and continued the SGLT2i because the patient had achieved adequate blood glucose control and his ideal body weight of <70 kg (Figure 5, black arrow).

**Figure 6:**
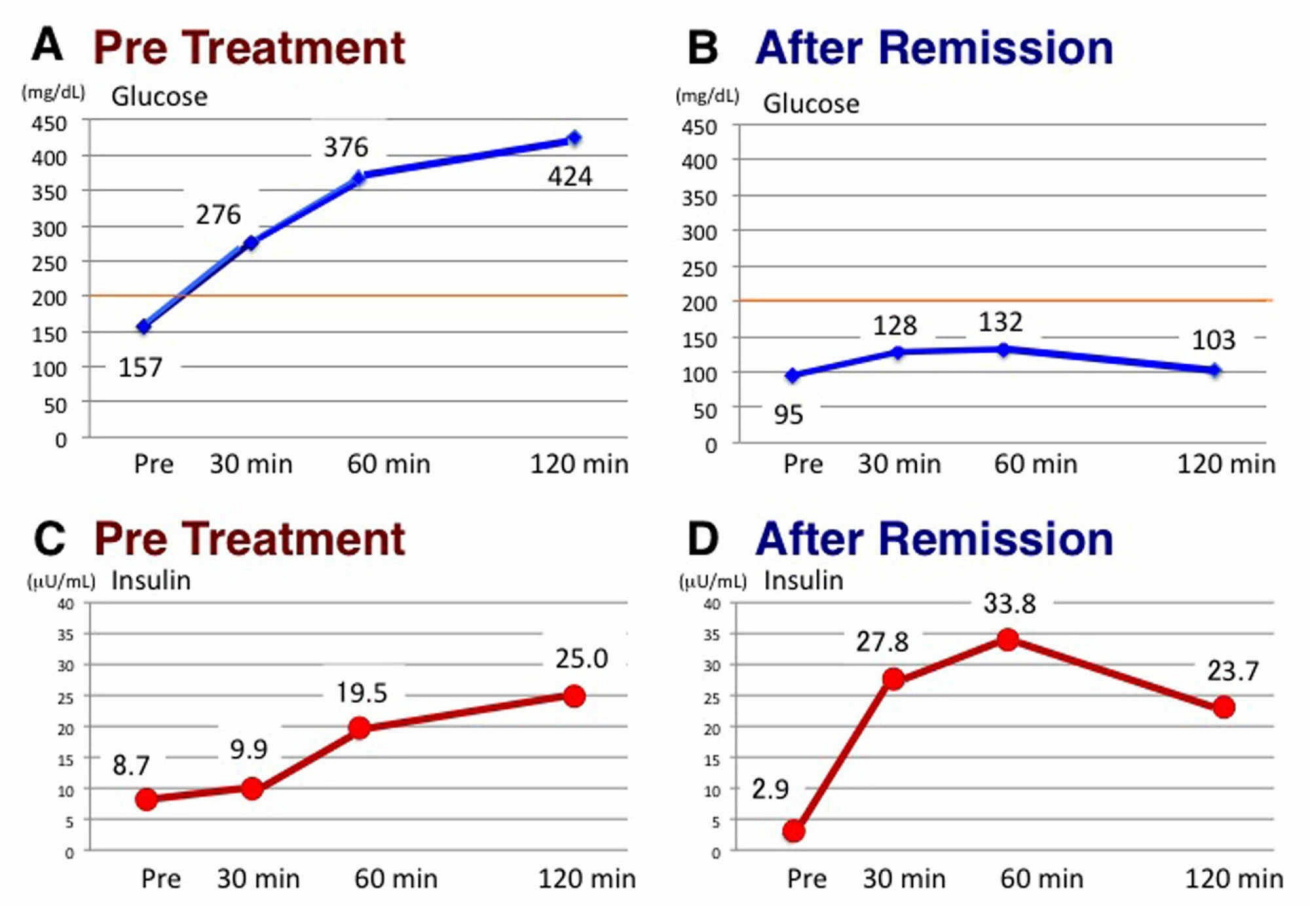
Changes in glucose and insulin concentrations in 75 g oral glucose tolerance test before and after remission of diabetes A: Glucose concentrations before therapy; B: Glucose concentrations after remission; C: Insulin concentrations before therapy; D: Insulin concentrations after remission

In December 2018, we began treating the patient with alternate-day administration of the SGLT2i, and in February 2019, we added a half-dose of metformin (500 mg/day) because his HbA1c increased from 5.2 to 5.3%. In April 2019, we stopped the SGLT2i and treated the patient with low-dose metformin alone. Finally, in June 2019, we discontinued all glucose-lowering medications and re-examined the patient at the outpatient clinic every other month. His glucose control and body weight were well-managed and remained within the normal ranges (Figure 5, gray arrows). Tables 3 and Figure 6 show the changes in the patient’s glucose metabolic and anthropometric parameters before treatment and after remission of T2DM for six months.

His glucose metabolic parameters nearly normalized, and the early phase of insulin secretion successfully recovered after an oral glucose load (Figure 6). Interestingly, abdominal computed tomography after T2DM remission showed that the volume of the pancreas had increased from 52.5 cm^3^ to 61.4 cm^3^. Bioelectrical impedance analysis using a body composition analyzer (InBody 770; InBody USA, Cerritos, CA) indicated almost no changes in his skeletal muscle mass and amount of body water during therapy [13]. However, his body fat mass had dramatically decreased. Furthermore, his visceral and subcutaneous abdominal fat areas had markedly decreased and his ectopic fat accumulation in the liver had normalized (Figure 4B-4D) [12]. No serious adverse events occurred during therapy.

## Discussion

The overall goal in the clinical treatment of T2DM is remission [10,14]. We experienced a case involving an overweight 43-year-old Japanese man who successfully achieved an approximately 20% reduction in body weight. He also achieved complete remission of his newly diagnosed T2DM after comprehensive therapy, including the administration of an SGLT2i.

T2DM is widely recognized as a chronic progressive disease that requires incremental lifelong hypoglycemic treatments [1]. T2DM has various pathogenic causes and is associated with several clinical conditions, including insulin resistance and pancreatic β-cell dysfunction and the disease course varies among patients [2-3]. The onset of T2DM is strongly associated with body weight gain and excess ectopic fat accumulation in the liver and pancreas [4-5]. Bariatric surgery leads to the remission of T2DM in patients with severe obesity [15]. However, bariatric surgery is an invasive therapy with risks of complications and is not available to all patients, particularly those with moderate obesity and without health insurance coverage for the surgery [15]. In 2018, Lean et al. reported that after a noninvasive intervention of a bodyweight management program with a low-calorie diet, patients with obesity and T2DM lost an average of 10 kg of body weight [14]. These authors also found that nearly half of the patients had reverted to a non-diabetic condition without the use of hypoglycemic medications [14]. This previous study shows the clinical possibility that weight management using a low-calorie diet can help patients with obesity achieve remission of T2DM. These findings also lend support to the widespread use of this weight reduction intervention in the routine care of patients with T2DM across general practice health services. Our patient achieved normal glucose concentrations after substantial bodyweight loss, mainly in fat weight, primarily by treatment with an SGLT2i and lifestyle intervention therapy without a low-calorie diet. We also recognized that the metformin monotherapy is generally not enough to control body weight as observed in this case. Metformin and integrated lifestyle modification, in addition to SGLT2i, played an important therapeutic role in the remission of T2DM. We were able to reduce the dosage of SGLT2i and, finally, achieve remission of T2DM by reducing the patient’s liver fat and recovering pancreatic β-cell function in daily clinical practice. Based on our experience, we propose the use of this treatment regimen to achieve remission of T2DM without a low-calorie diet in the current era of SGLT2i therapy.

In the early phase of T2DM, we initially provide patients with instructions regarding lifestyle modification by diet and exercise therapy to reach an appropriate body weight and correct excessive calorie intake [2]. We then add glucose-lowering pharmacotherapy if adequate glycemic control has not been achieved [2]. We recognize that the successful continuation of dietary and exercise therapy is important to achieve remission of T2DM. Some patients with T2DM can maintain good glycemic control by only diet and exercise therapy, and some patients completely discontinue all hypoglycemic drugs, resulting in remission of T2DM [3,10]. In actual daily clinical practice, the provision of specific and personal direction and instruction for lifestyle improvement tends to be insufficient because clinical staff members lack the time to adequately care for patients. Additionally, health care insurance does not cover exercise therapy for T2DM in Japan. Remission of T2DM can easily regress to overt T2DM, requiring hypoglycemic medications.

In the clinical setting, SGLT2i therapy can lead to body weight loss (mainly by fat reduction without intensive dietary management, such as a low-calorie diet) soon after starting drug administration [7,13]. SGLT2i therapy can thus serve as an effective and less stressful weight reduction strategy in overweight or obese patients with T2DM. The possibility of achieving a desirable body weight through weight reduction therapy can help maintain the patient’s motivation, leading to sustainable weight reduction in the current era of SGLT2i therapy. SGLT2i-induced weight reduction and normalization might spontaneously promote a patient’s change in behavior to successfully maintain daily diet and exercise therapy. We clinicians should keep the importance of such a comprehensive treatment strategy, including SGLT2i, in mind.

Theoretically, SGLT2i-induced urinary caloric loss can be sustained even after the improvement of glycemic control. Additionally, SGLT2i-induced weight loss is sustained because the SGLT2i can continue to accelerate the excretion of glucose into the urine in patients without T2DM [9,16]. In most patients, however, the amount of SGLT2i-induced weight loss is approximately 2 kg to 3 kg, and this weight loss plateaus within six months [17-18]. Weight reduction cannot be achieved overnight; persistent efforts and steady continuation of lifestyle modification therapy are required. When we introduced SGLT2i-induced weight reduction therapy in our Diabetes Care Center, we advised overweight patients with T2DM to measure and record their body weight twice a day (morning and night). We experienced that this advice was practically effective even in the clinical situation of compensated eating induced by SGLT2i treatment. The findings in the present case support the use of a weight reduction program to obtain healthy normal body weight and remission of T2DM on an outpatient basis. Daily recording of body weight provides a clearer understanding of the patient’s clinical condition and helps sustain adequate diet therapy and maintain the patient’s motivation toward obtaining their ideal weight.

In the Japanese population, the risk of developing T2DM and insulin resistance increases at a BMI of ≥ 23 kg/m^2^ [19]. Therefore, a decrease in body fat induced by weight reduction therapy might help improve insulin resistance in mildly to moderately overweight Japanese patients with T2DM. Insulin resistance is a fundamental pathogenic condition of T2DM not only in patients with severe obesity who are clinical candidates for bariatric surgery but also in overweight patients with T2MD in Japan [5,20]. Comprehensive anti-diabetic and weight reduction therapy, including SGLT2i administration, might remove the excessively accumulated fat [7,9]. This could lead to an improvement in insulin resistance, fatty liver, and glucose metabolism [12]. The findings from the present case suggest that an integrated anti-diabetic and weight reduction strategy can be developed with adequate diet and exercise therapy, including the administration of an SGLT2i, in overweight patients at the early stage of T2DM. In the future, it will be interesting to consider the contribution of lifestyle modification therapy and SGLT2i to the improvement and remission of T2DM.

## Conclusions

The overall goal in the treatment of T2DM is remission. We herein report a case involving an overweight 43-year-old man who completely recovered from T2DM after comprehensive therapy, including the administration of an SGLT2i and metformin. We recommend that clinicians attempt a weight reduction strategy to achieve remission of T2DM in overweight patients. We propose the usefulness of an SGLT2i for achieving a normal body weight and remission of newly diagnosed T2DM in a real-world clinical situation.
